# The outcomes of total hip arthroplasty in developmental dysplasia of hip versus osteoarthritis: a systematic review and meta-analysis

**DOI:** 10.1007/s00590-023-03635-6

**Published:** 2023-07-06

**Authors:** Loay A. Salman, Osama Z. Alzobi, Abdallah Al-Ani, Ashraf T. Hantouly, Mohammed Al-Juboori, Ghalib Ahmed

**Affiliations:** 1 Orthopedics Department, Surgical Specialty Center, Hamad General Hospital, Hamad Medical Corporation, PO Box 3050, Doha, Qatar; 2 Office of Scientific Affairs and Research, King Hussein Cancer Center, Amman, Jordan

**Keywords:** Developmental dysplasia of the hip, Osteoarthritis, Total hip arthroplasty, Revision, Outcome

## Abstract

**Purpose:**

This systematic review and meta-analysis aimed to compare the outcomes of total hip arthroplasty (THA) in patients with developmental dysplasia of the hip (DDH) and those with osteoarthritis (OA).

**Methods:**

Four databases were searched from inception till February 2023 for original studies that compared the outcomes of THA in DDH and OA. The primary outcome was the revision rate; the secondary outcomes were dislocation and failure modes (i.e. aseptic loosening, PJI, instability, and periprosthetic fractures), hospital stay and costs. This review was conducted as per PRISMA guidelines, and the risk of bias was assessed using the Newcastle–Ottawa scale.

**Results:**

A total of 9 observational studies with 575,255 THA (469,224 hips) were included, with a mean age of 50.6 years and 62.1 years for DDH and OA groups, respectively. There was a statistically significant difference in revision rate between DDH and OA patients in favour of OA (OR, 1.66; 95% CI 1.11–2.48; *p*-value, 0.0251). However, dislocation rate (OR, 1.78, 95% CI 0.58–5.51; *p*-value, 0.200), aseptic loosening (OR, 1.69; 95% CI 0.26–10.84; *p*-value, 0.346) and PJI (OR, 0.76; 95% CI 0.56–1.03; *p*-value, 0.063) were comparable across both groups.

**Conclusion:**

A higher revision rate following total hip arthroplasty was associated with DDH compared with osteoarthritis. However, both groups had similar dislocation rates, aseptic loosening and PJI. Consideration of confounding factors, such as patient age and activity level, is crucial when interpreting these findings.

**Level of evidence:**

III.

**Trial registration:**

PROSPERO registration: CRD42023396192.

## Introduction

Total hip arthroplasty (THA) is a frequently performed surgical intervention to alleviate hip pain and dysfunction when non-operative measures have failed. There are estimated to be more than 500,000 annual THA cases in the USA alone [1]. Primary hip osteoarthritis (OA) and developmental dysplasia of the hip (DDH) are the two most common etiologists for THA [2–6]. DDH is a spectrum of diseases that ranges from a shallow acetabulum to a complete dislocation of the femoral head. This condition results in acetabular and femoral changes that disrupt the normal hip anatomy and biomechanics, making DDH the leading cause of secondary hip osteoarthritis requiring THA. In addition, DDH is responsible for 20% of all THA in patients younger than 50 years [7], thus, posing a significant burden on patients and the entire healthcare system.

Given the complexity of DDH and associated challenges, the long-term consequences, potential complications, and success rate of THA in such cases remain debatable. While some studies showed satisfactory and good clinical outcomes, others reported inferior long-term results and higher revision rates [8].

To the best of our knowledge, no previous systematic reviews have directly assessed the long-term outcomes and safety of THA in DDH versus osteoarthritis. Therefore, this study aimed to review and compare the clinical outcomes of THA in patients with DDH to those with hip OA. This meta-analysis hypothesises that THA in patients with DDH has inferior clinical outcomes and higher complication and revision rates.

## Methods

This systematic review adhered to the guidelines provided by the Preferred Reporting Items for Systematic Reviews and Meta-Analyses (PRISMA) [9]. The review protocol was registered in advance on the International Prospective Register of Systematic Reviews (PROSPERO) with the Registration Number CRD42023396192.

### Search strategy

A comprehensive search was conducted across several databases, including PubMed/Medline, Ovid, Google Scholar, and the Cochrane Library. The search encompassed the entire available literature up until February 2023, using a combination of specific keywords and their variations. The keywords used were “Total hip replacement” OR “Total hip arthroplasty”, “Developmental dysplasia of the hip”, “Osteoarthritis”, and “Outcomes.” The search results were initially screened by two authors independently, who assessed the relevance based on each article’s title and/or abstract. In case of disagreements, a meeting involving a third, more senior author was held to resolve any conflicts. A thorough review of the full-text articles that met the eligibility criteria was conducted following the initial screening. Additionally, the reference lists of these selected articles were manually examined to ensure that all relevant studies were included in the analysis.

### Outcomes of interest

The primary outcome was the revision rate, defined by the National joint registry consensus as “Any operation performed to add, remove or modify one or more components of a joint replacement” [10]. The number of dislocations, modes of failure (i.e. aseptic loosening, PJI, instability, and periprosthetic fractures), hospital stay and costs were used as secondary outcomes of interest.

### Eligibility criteria

#### Inclusion criteria

1. All original comparative, RCTs and observational studies reporting the outcome of THR in Hip dysplasia or primary OA.
2. Studies with a minimum follow-up period of 1 year.
3. All types of THR prosthesis designs.

#### Exclusion criteria

1. Studies with different indications for THR other than hip dysplasia or OA
2. Noncomparative or not reporting outcomes or failures by subgroups (i.e. DDH vs OA)
3. Review articles, cross-sectional, preclinical studies, case series and reports
4. Studies with incomplete or unextractable data
5. Studies published in languages other than English

### Data extraction and items

Two independent reviewers used a pre-defined Microsoft Excel data collection sheet to extract relevant data. The extracted data encompassed various demographic aspects, such as the surname of the first authors, study year, design and location, mean patient age, number of participants and hips, age, type of total hip replacement (THR) (including cementless and cemented), type of prosthesis and bearings used, mean follow-up period, number of revisions, complications, modes of revisions, hospital stays, costs, statistical tests employed, and the conclusion of each study.

### Qualitative assessment (Risk of bias)

Two authors assessed the methodological quality of the included studies using the Newcastle–Ottawa tool, which comprises three key domains; patient selection, comparability, and outcomes [11, 12]. A higher overall score indicates a lower risk of bias; a score of 5 or less (out of 9) corresponds to a high risk of bias.

### Quantitative analysis

A meta-analysis of eligible studies using R software (version 4.0.2, R Core Team, Vienna, Austria, 2020), particularly the meta package (i.e. forest_meta and metabin), was performed. Odds ratios (OR) and their associated 95% confidence intervals were expressed for dichotomous variables (e.g. rate of revisions). Heterogeneity among effect sizes was evaluated using the I-squared statistic. Definitions for heterogeneity were adapted from the Cochrane Handbook (> 25% mild, 25–50% moderate, > 50% severe). Due to the high heterogeneity for the dichotomous variables, a random-effects model was utilized. Both a funnel plot and Egger’s test of asymmetry were used to assess publication bias.

## Results

### Study selection

**Fig. 1:**
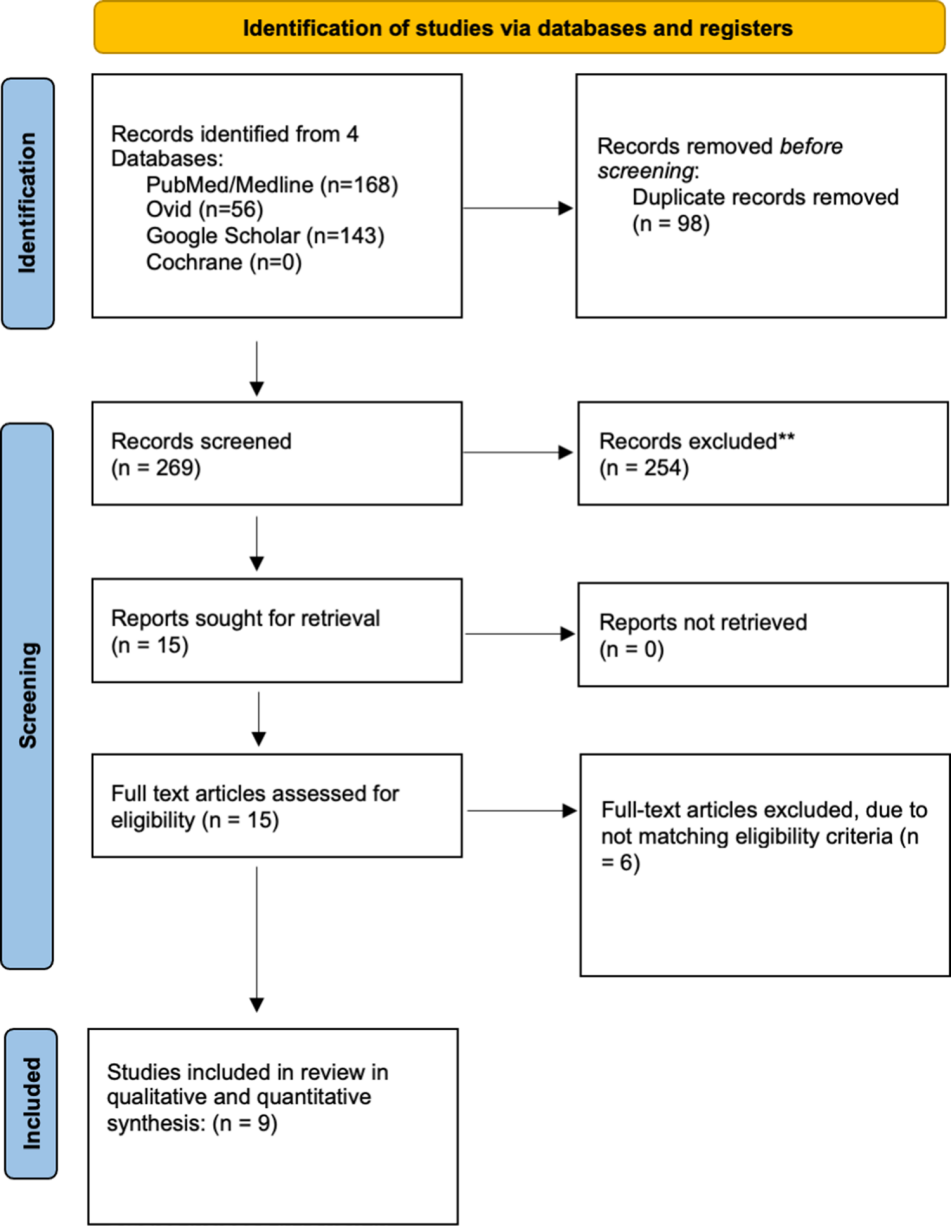
PRISMA flow diagram of record identification, screening and selection in meta-analysis

Rayyan AI website was used to manage the literature search results [13]. Searching the databases yielded 367 articles, and after removing 98 duplicates, 269 records were screened by title and abstracts, of which 254 were excluded. A total of 15 papers were eligible for a full-text review. As a result, 9 studies met the eligibility criteria and were included in the qualitative and quantitative synthesis. The PRISMA flowchart is displayed in Fig. 1.

### Study characteristics

A total of 9 articles investigating the impact of total hip arthroplasty (THA) on patients with developmental dysplasia of the hip (DDH) and osteoarthritis (OA) were included. The included studies were published during the time period from 2008 to 2022. Included publications were primarily from the United States of America (*n* = 3), Denmark, Norway and Sweden (*n* = 3), New Zealand (*n* = 2), and Iran (*n* = 1). These studies were entirely cohort-based, of which 8 were retrospective and 1 was prospective (Table 1).

**Table 1:** A summary of baseline study characteristics, LoE: level of evidence, FU (Y): follow-up in years

References	Design, LoE	Country	No. hips	THA type	FU (Years)	Age (DDH/OA)	Gender% (F:M)	Data source
Engesæter [2]	Cohort, II	Norway	66,909	Cemeted, Cementless	15	56/71	DDH: 75.9% F, 24.1% M / OA: 69.1% F, 30.9% M	Norwegian Arthroplasty Register (NAR)
Thillemann [17]	Retrospective, III	Denmark	56,087	Cemented, Cementless, Hybrid	4.6	Age Subgroups	DDH: 1069 F, 386 M / OA: 30,296 F, 23,398 M	Danish Hip Arthroplasty Registry
Boyle [24]	Retrospective, III	New Zealand	41,794	Cemented, Cementless, Hybrid	10	49.3/67.6	DDH: 74% F, 26% M / OA: 52% F, 48% M	New Zealand National Joint Registry
Boyle [18]	Retrospective, III	New Zealand	1054	NR	Min.1 year	56.6/79.2	DDH: 69.7% F, 30.3% M / OA: 50.9% F, 49.1% M	Regional joint registry records (5-year data)
Engesæter [16]	Retrospective, III	Denmark, Norway, and Sweden	300,503	Cemented, Cementless, Hybrid	DDH: 6.4 / OA: 5.9	56/69.3	DDH: 72.5% F, 27.5% M / OA: 59.6% F, 40.4% M	NARA Registry
Ashraf [7]	Retrospective, III	USA	1383	NR	8	49 /54	DDH: 81% F, 19% M / OA:74% F, 26% M	OCHEUD / Institutional Data Registry
Aggarwal [1]	Retrospective, III	USA	836	NR	Min. 1 year	54.4 (+ -12.6)/ 64 (+ -11.9)	DDH: 77.4% F, 22.6% M / OA: 59.6% F, 40.6% M	Institutional electronic medical records
Siddiqi [8]	Retrospective, III	USA	115,769	NR	Min. 1 year	Age Subgroups	DDH: 73.5% F, 26.5% M / OA: 55.3% F, 40.7% M	National Surgical Quality Improvement Program database
Mortazavi [19]	Retrospective, III	Iran	368	NR	DDH: 40.4 ± 18.2 / OA: 42.3 ± 16.4	42.23 +—6.07 / 46.86 ± 15.49	DDH: 88.82% F, 11.18% M / OA: 55.43% F, 44.57% M	Tehran University of Medical Sciences

A total of 575,255 THA procedures were recorded across the included studies. A total of 469,224 hips were examined. Altogether, 23,072 hips with DDH were examined while 445,894 hips with OA were pooled. The mean age for participants with DDH was 50.6 years, while participants with OA had a mean age of 62.1 years. The pooled number of participants were followed up for anywhere between 4.6 and 42.3 years.

### Quality assessment (risk of bias and level of evidence (LoE))

Following OCEBM criteria [14], one study was level 2b and eight were level 3a (Table 1), with an overall grade B of recommendation assigned to the review [15]. The Newcastle–Ottawa scores of all 9 observational studies ranged from 6 to 8, with an average of 7 ± 0.67, indicating a low overall risk of bias. A summary of the qualitative assessment, using the Newcastle–Ottawa scale, is displayed in Table 2.

**Table 2:** Risk of bias was assessed using the Newcastle–Ottawa scale. A higher overall score indicates a lower risk of bias; a score of 5 or less (out of 9) corresponds to a high risk of bias

References	Selection	Comparability	Outcome	Total score
Aggarwal [1]	****	*	**	7
Ashraf [7]	****	*	**	7
Boyle [24]	****	*	**	7
Boyle [18] (Functional)	****	*	**	7
Engesæter [2]	****	*	***	8
Engesæter [16]	****	*	***	8
Mortazavi [19]	****	0	**	6
Siddiqi [8]	****	0	**	6
Thillemann [17]	****	*	**	7

### Outcome results

**Fig. 2:**
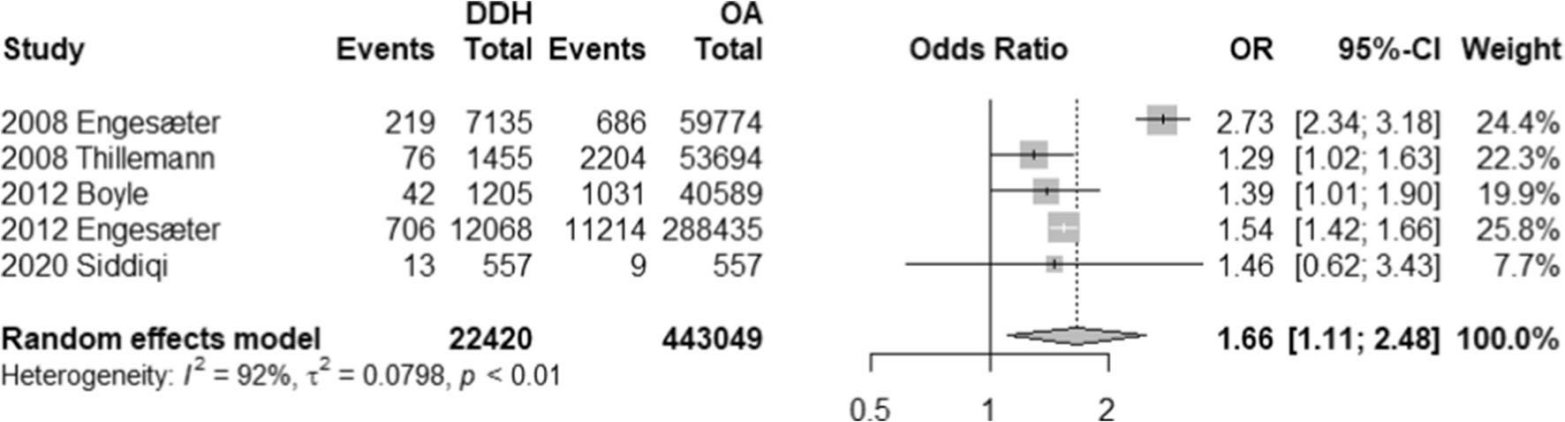
Forest plot comparison of the overall revision between DDH and OA patients. CI confidence interval. OR: Odds ratio

**Fig. 3:**
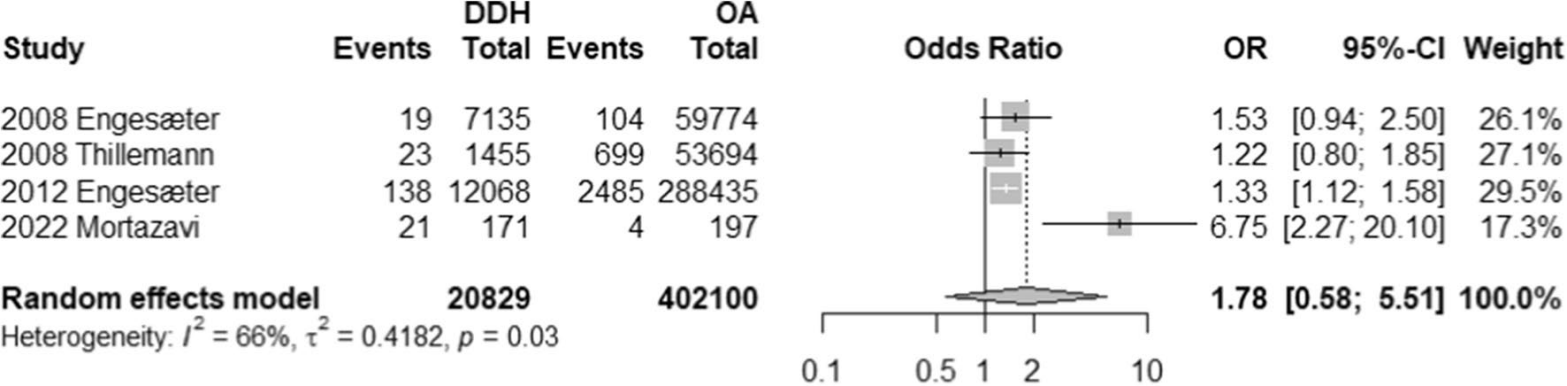
Forest plot comparison of the overall dislocation between DDH and OA patients. CI confidence interval. OR: Odds ratio

The impact of THA on DDH and OA were examined through a number of outcome measures including number of revisions, number of dislocations, and modes of treatment failure (i.e. aseptic loosening, PJI, instability, and periprosthetic fractures). In terms of revisions, patients with DDH are 1.66 times more likely to have revisions than their OA counterparts (OR, 1.66; 95% CI 1.11–2.48; *p*-value, 0.0251) (Refer to Fig. 2). On the other hand, a total of 4 studies reported on number of dislocations and demonstrated that patients with DDH are 1.78 times more likely to experience dislocations; however, such difference is statistically insignificant (OR, 1.78, 95% CI 0.58–5.51; *p*-value, 0.200) (Refer to Fig. 3).

**Fig. 4:**
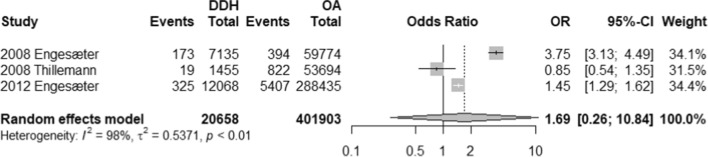
Forest plot comparison of the aseptic loosening between DDH and OA patients. CI confidence interval. OR: Odds ratio

**Fig. 5:**
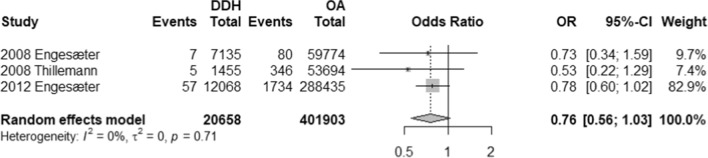
Forest plot comparison of periprosthetic joint infection between DDH and OA patients. CI confidence interval. OR: Odds ratio

With respect to modes of failure, only aseptic loosening and PJI were eligible for quantitative analysis reported by 3 studies each. Patients with DDH were 1.69 more likely to have aseptic loosening (OR, 1.69; 95% CI 0.26–10.84) and were 0.76 times less likely to have PJI (OR, 0.76; 95% CI 0.56–1.03) (Refer to Figs. 4 and 5). However, both rates were statistically insignificant (*p*-value, 0.346 and 0.063, respectively). In addition, no studies reported on instability while only one study reported on periprosthetic fractures (PPF). Thillemann et al. (2008) reported 4 and 109 PPF incidents among 1455 patients with DDH and 53,694 patients with OA, respectively.

Three studies reported on hospital stay and hospital costs associated with THA (Ashraf 2014, Aggarwal 2019, and Siddiqi 2020). Hospital stays for patients undergoing THA ranged from 3 to 11 days. Moreover, hospital costs ranged from 16,949$ to 28,207$ for patients with DDH, and from 16,485$ to 27,078$ for patients with OA.

## Discussion

The most significant findings of this systematic review and meta-analysis were that the revision rate was significantly higher following THA for DDH than for primary OA. Another important finding was that patients with DDH were more likely to have dislocations and aseptic loosening; however, such differences were statistically insignificant. Additionally, Hospital stay and hospital costs were comparable.

### Revision

This study found a significant increase in revision procedures among the DDH group following their initial surgery compared to THA for primary OA. This finding is consistent with some previous studies. Engesæter12 et al. [16] have postulated that patients with DDH have an increased overall risk of revision within the first six months postoperatively in comparison to patients with OA. However, the higher revision rate was not sustained, and there was no significant difference after the first six months. The authors attributed this finding to the fact that dislocation was significantly higher in DDH than for OA within the first six months [16]. The authors have also reported no difference in the risk of revision due to aseptic loosening or infection [16].

Thillemann et al. [17] and Boyle et al. [18] reported a higher rate of revision favouring OA, but the difference was not statistically significant. Meanwhile, Siddiqi et al. [8] reported comparable results across both groups. The reasons for these findings are not clearly defined, and caution should be exercised when interpreting these results due to significant heterogeneity among the studies. Factors such as pooling mild and severe dysplasia groups likely underestimate the risks of revision associated with THA for severe dysplasia and may have played a role in the differences observed between studies [2]. Additionally, DDH patients are often younger and more active than those with primary OA and may have higher expectations for their postoperative function and activity levels. This may lead to revision surgery being offered more readily in this population, compared to older patients with primary OA who may have multiple co-morbidities that could make revision surgery less feasible or desirable.

### Dislocations

Four studies were included in this meta-analysis that investigated the dislocation rate between both cohorts. The cohort undergoing THA for DDH demonstrated an overall statistically insignificant higher dislocation rate than OA patients. This increased risk of dislocation in THA after DDH is probably to be expected since DDH patients often have extreme anteversion of the proximal femur, which governs the anteversion of the femoral component and thereby increases the risk of anterior dislocation. Mortazavi et al. reported that the dislocation rate was significantly higher in the DDH group than in the primary OA group [19]. Furthermore, a univariate analysis in the same study indicated an increase in the dislocation rate in relation to the severity and grade of DDH. Similarly, a significant increase in dislocation rate was also observed in two other studies [2, 16]. This can be due to a dysplastic acetabulum, a narrow femur, shortening, rotational deformity, and previous surgeries can further contribute to the risk of dislocation. The altered anatomy can lead to a technically challenging THA with a higher likelihood of dislocation. Further, it is essential to consider the risk of dislocation as a significant factor when assessing the overall revision rate.

### Aseptic loosening

The cohort undergoing THA for DDH revealed a statistically insignificant higher aseptic loosening than the OA group. While earlier studies have shown a higher risk of aseptic loosening of the acetabular component following THA surgery in patients with DDH [20, 21], it is essential to note that the difference was not statistically significant in this meta-analysis. This suggests that the risk of aseptic loosening may have decreased over time as improvements in surgical technique and prosthetic design have been made.

The inclusion of a large sample size, long follow-up periods, and high-quality studies with low risk of bias were key strengths that bolstered the external validity and generalizability of our findings. Additionally, the incorporation of various total hip replacement (THR) prosthesis designs (hybrid, cementless, and cemented) further contributed to the robustness of our results.

However, it is important to acknowledge several limitations. Firstly, the analysis of the developmental dysplasia of the hip (DDH) cohort encompassed a range of presentations, from dysplasia to dislocation. Conducting subgroup analyses based on specific DDH morphological severity would have mitigated the heterogeneity of this condition and its potential impact on the overall outcome of THA. Unfortunately, limited studies and inconsistent reporting of DDH in the literature hindered the feasibility of this approach.

Another potential limitation was the inadequate reporting of factors that could influence the outcomes of THR, such as implant type and surgical factors [22, 23]. Moreover, future research should prioritize prospective studies to better control for these confounding factors and to evaluate this issue in a statistically robust manner.

## Conclusion

This study demonstrated a significantly higher revision rate in patients with DDH following total hip arthroplasty compared to patients with primary osteoarthritis. However, dislocation rates, aseptic loosening and PJI were comparable. This finding should be applied in context given the heterogeneity of patients and confounding factors.

## Data Availability

Not applicable. However, happy to provide access to any statistical data (coding) upon request.
